# Racial disparities and factors associated with pregnancy in kidney transplant recipients in the United States

**DOI:** 10.1371/journal.pone.0220916

**Published:** 2019-08-09

**Authors:** Silvi Shah, Annette L. Christianson, Prasoon Verma, Karthikeyan Meganathan, Anthony C. Leonard, Daniel P. Schauer, Charuhas V. Thakar

**Affiliations:** 1 Division of Nephrology Kidney C.A.R.E. Program, University of Cincinnati, Cincinnati, Ohio, United States; 2 Department of Biomedical Informatics, University of Cincinnati, Cincinnati, Ohio, United States; 3 Division of Neonatology, Cincinnati Children’s Hospital and Medical Center, Cincinnati, Ohio, United States; 4 Department of Family and Community Medicine, University of Cincinnati, Cincinnati, Ohio, United States; 5 Department of Internal Medicine, University of Cincinnati, Cincinnati, Ohio, United States; 6 Division of Nephrology, VA Medical Center, Cincinnati, Ohio, United States; Chulalongkorn University, THAILAND

## Abstract

**Background:**

Although kidney transplant improves reproductive function in women with end-stage kidney disease (ESKD), pregnancy in kidney transplant recipients’ remains challenging due to the risk of adverse maternal and fetal outcomes.

**Methods:**

We evaluated a retrospective cohort of 7,966 women who were aged 15–45 years and received a kidney transplant between January 1, 2005 and December 31, 2011 from the United States Renal Data System with Medicare as the primary payer for the entire three years after the date of transplantation. Unadjusted and adjusted rates of pregnancy in the first three post-transplant years were calculated, using Poisson regression for the adjustment. Factors associated with pregnancy, including race, were examined using logistic regression.

**Results:**

Overall, 293 pregnancies were identified in 7966 women. The unadjusted pregnancy rate was 13.8 per thousand person-years (PTPY) (95% confidence interval (CI), 12.3–15.5). Pregnancy rates were roughly constant in the years 2005–2011 except in 2005 and 2010. The rate of pregnancy was highest in Hispanic women (21.4 PTPY; 95% CI, 17.2–26.4) and Hispanic women had a higher likelihood of pregnancy as compared to white women (OR, 1.56; CI, 1.12–2.16). Pregnancy rates were lowest in women aged 30–34 years and 35–45 years at transplant, and women aged 30–34 years and 35–45 years at transplant were less likely to ever become pregnant during the follow-up (odds ratio [OR], 0.69; CI, 0.49–0.98 and OR, 0.14; CI 0.09–0.21 respectively) as compared to women aged 25–29 years at time of transplant. Women had higher rates of pregnancy in the second and third-year post-transplant (16.0 PTPY, CI 13.2–19.2 and 16.9 PTPY, CI 14.0–20.4) than in the first-year post-transplant (9.0 PTPY, CI 7.0–11.4). In transplant recipients, pregnancy was more likely in women with ESKD due to cystic disease (OR, 2.42; CI, 1.02–5.74) or glomerulonephritis (OR, 2.14; CI, 1.07–4.31) as compared to women with ESKD due to diabetes.

**Conclusion:**

Hispanic race, younger age, and ESKD cause due to cystic disease or glomerulonephritis are significant factors associated with a higher likelihood of pregnancy. Pregnancy rates have been fairly constant over the last decade. This study improves our understanding of factors associated with pregnancy in kidney transplant recipients.

## Introduction

Childbearing is an integral part of women’s lives. Kidney disease leads to dysregulation of the hypothalamic-gonadal axis, menstrual cycle abnormalities, and impaired fertility [1, 2]. Pregnancy in women on dialysis is not common and the reported rates of conception range from <1% to 7% [3]. Fertility restores quickly in a women with end-stage kidney disease (ESKD) after they receivea kidney transplant and a kidney allograft is able to adapt to normal physiological changes of pregnancy [4]. However, managing pregnancy in a kidney transplant recipient is difficult due to the adverse effects of immunosuppressive drugs, the risk of worsening of kidney function and maternal and fetal complications [5–8].

Family planning counseling is an important part of the transplant evaluation process and post-transplant clinical care [9]. Although pregnancy is common in women with functioning kidney transplants, little is known about pregnancy rate changes during the past ten years in the United States [10]. No study has examined the extent to which factors like race, ESKD cause, and time of conception are associated with pregnancy in the kidney transplant recipients. More importantly, current knowledge regarding pregnancy in kidney transplant patients is limited to small numbers from single-center studies, and reporting bias from surveys and voluntary registries [11–14].

Since women of childbearing age comprise a substantial proportion of kidney transplant recipients, it becomes imperative to examine the incidence of pregnancy in the recent decade. We used the national ESKD registry, the United States Renal Data System (USRDS), to determine the rates of pregnancy in women with kidney transplant from 2005–2011 in the first three post-transplant years by race, age, year of conception, and cause of ESKD. The present study involves the largest cohort of kidney transplant recipients in the United States, which is not a voluntary registry, and examines the factors associated with pregnancy, including race.

## Methods

### Design, setting, and participants

**Fig 1:**
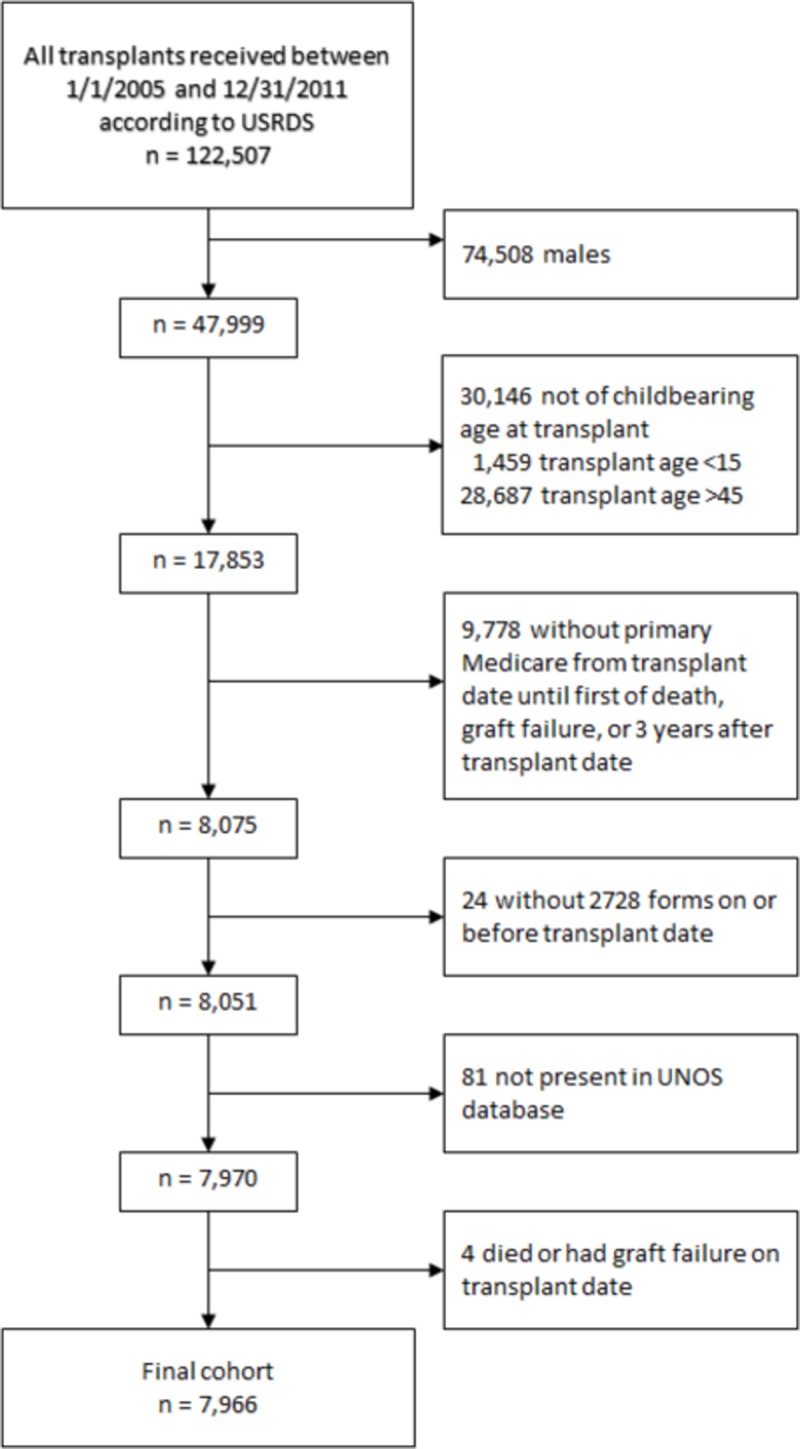
Cohort selection flow diagram.

We performed an observational study to determine the incidence of pregnancy in women with kidney transplant in the first three years post-transplant in the United States. Using the USRDS, we evaluated women who were aged 15–45 years and received a kidney transplant between January 1, 2005 and December 31, 2011 and had primary Medicare claims data for the entire three years post-transplant. Patients were followed until the first of graft failure, death, or three years after the transplant date. The USRDS payer history file was used to obtain information on insurance coverage for the study period. We excluded patients with no baseline CMS-2728 form and with missing data on race. Subsequent kidney transplants within the study period were not considered. **Fig 1**shows derivation of the study cohort. The study was deemed exempt by the University of Cincinnati Institutional Review Board committee because the data were de-identified.

### Outcome

The primary outcome of interest was the incidence of pregnancy during the time period from transplant to the earliest of three years post-transplant. The International Classification of Diseases, 9th Revision, Clinical Modification (ICD-9-CM) codes, Current Procedural Terminology, 4th Revision (CPT-4) codes, and the Diagnostic Related Group (DRG) codes were used to identify pregnancy-related clinical encounters from the inpatient and outpatient Medicare claims. The specificity of this code-based method for determining the occurrence of pregnancy has been validated in the prior studies [15, 16]. Codes that indicated the end of pregnancy were more informative about timing and outcome, so were used first to identify the occurrence of pregnancy **(S1 Table).** Pregnancy outcomes were categorized into live births, stillbirths, abortions (includes spontaneous and therapeutic), ectopic/trophoblastic pregnancies, and unknown outcomes [15, 17]. Next, codes that identified the occurrence of pregnancy, but did not indicate its completion, were grouped into additional pregnancies **(S1 Table).** Conception dates were estimated from the outcome-specific estimates of gestational age. We used an interval of twenty-four weeks to determine the ends of two pregnancies that resulted in deliveries, twenty weeks between an early loss and a subsequent delivery, ten weeks between a delivery and a subsequent early loss, and six weeks between two early losses. We adjusted the start dates for pregnancies that resulted in delivery by a maximum of eight weeks in case of overlap and start dates for pregnancies that resulted in an early loss by a maximum of two weeks.

### Study covariates

The USRDS transplant file was used to obtain information on age, sex, donor type (living or deceased), transplant date, and graft failure date. The USRDS patient file was used to obtain information on the history of kidney transplant, date of death, and ESKD networks classified into geographical regions of midwest, northeast, south, and west [18]. The CMS-2728 form was used to obtain information on age, race (Asian, black, Hispanic, Native American, and white), comorbidities (diabetes, hypertension, and congestive heart failure), and the cause of ESKD (diabetes mellitus, hypertension/large vessel disease, malignancy, cystic/hereditary, glomerulonephritis, secondary glomerulonephritis/vasculitis, interstitial nephritis/pyelonephritis, and others) [19]. The USRDS treatment history file was used to obtain information on the duration of dialysis prior to transplant (<1 year, 1–3 year, and > 3 years). Glomerular filtration rate (GFR) was calculated from serum creatinine values from six months after transplant obtained from the United Network for Organ Sharing (UNOS) follow-up file, using the Modification of Diet in Renal Disease equation, and reported in ml/min/1.73 m^2^. Information on immunosuppression at transplant was obtained from the linked UNOS registry data. We created groups for the covariates based on clinical relevance, and patients with unavailable information were categorized into a “missing” group for that covariate. Pregnancies were categorized by the interval between kidney transplant and conception as first, second, and third year post- transplant.

### Statistical analysis

Summary statistics are presented as percentages for categorical data and mean ± standard deviation (SD) for continuous variables. Differences between the groups were tested with chi-square tests for categorical variables and t-tests or one-way ANOVAs for continuous variables. Statistical significance was set at a two-tailed p-value of 0.05, unadjusted for multiple tests. We calculated the unadjusted rates of pregnancy in the first three years post-transplant by age at transplant, race, and year of conception related to transplant. Rates were expressed as the number of pregnancies per 1000 person-years (PTPY). We then used the Poisson regression model to calculate adjusted rates of pregnancy. Exposure time began on the date of transplant and ended with the first of the following events: death, graft loss, or three years post-transplant. The model contained year of transplant, age at transplant, and race as independent variables, as well as an offset term with log exposure time. A deviance scale parameter was included to correct for underdispersion. We calculated adjusted rates and the 95% CIs using the observed marginal distributions. Because 19.5% of pregnancies were identified only using codes available at the end of the pregnancy, we conducted a sensitivity analysis in which conception dates were cut off 40 weeks before the end of follow up. This analysis de facto excluded women who died or had graft failure before they could have been known to be pregnant **(S2 Table).** A second sensitivity analysis excluded women after they turned 46 years old, to determine whether rates in the oldest group and the later conception years were being underreported due to women ceasing to be of childbearing age **(S2 Table).**

Multivariable logistic regression was used to determine the factors associated with pregnancy. Multivariable models were non-parsimonious and included the following covariates: maternal age at transplant, race, donor type, transplant year, ESKD cause, immunosuppression at the time of transplant, GFR at six months post-transplant, duration of dialysis before transplant, and comorbidities. Two additional sensitivity analyses were performed: 1) deaths and graft failures removed from the model, and 2) women with missing creatinine values removed from the model **(S3 Table).** All data were analyzed using SAS version 9.4 (SAS Institute, Cary, NC).

## Results

### Baseline demographics and clinical characteristics

Overall, 293 pregnancies were identified in 7,966 women of child bearing age in the three years following kidney transplant. The mean age of the study population at transplant was 33±8 years. White women comprised the largest racial group (39.9%), and most women received deceased donor kidney transplants (71.7%). The mean GFR six months after transplantation was 65.2±24.8, with 42% having a GFR < 60. About half (51.7%) had received dialysis for at least 3 years prior to receiving a kidney transplant. The majority received tacrolimus, mycophenolate, and prednisone as immunosuppression at the time of transplant. Glomerulonephritis was the most frequent cause of ESKD (41.8%), while only 18.5% had their ESKD attributed to diabetes. Compared to those with no pregnancies, women with pregnancies had lower rates of diabetes, while rates of hypertension and congestive heart failure were not significantly different. Higher proportions of women who had pregnancies had received living donor transplants, had a GFR ≥ 60 six months after transplantation, and received dialysis for less than three years prior to transplantation. Region, prior transplant status, and graft failure during follow-up period were not different for the two groups, but women who became pregnant were less likely to die during the follow-up period **(Table 1)**. This motivated our sensitivity analysis removing deaths and graft failures from the logistic regression model **(S3 Table).**

**Table 1:** Baseline characteristics of women of childbearing age separated by pregnancy during the follow-up period.

Baseline Characteristics of Women	All (n = 7,966)	Pregnant during followup (n = 249)	Not pregnant during followup (n = 7,717)	P Value
Age at transplant (years)*	33 (8)	27 (6)	34 (8)	<0.0001
	** **			<0.0001
15–19	6.8	10.8	6.7	
20–24	8.3	19.3	7.9	
25–29	13.9	28.9	13.4	
30–34	18.8	26.1	18.6	
35–45	52.2	14.9	53.4	
Race				<0.0001
Black	33.1	28.9	33.2	
Hispanic	18.7	29.7	18.3	
White	39.9	36.1	40.1	
Other/unknown	8.3	5.2	8.4	
Donor type				0.0363
Living	28.3	34.1	28.1	
Deceased	71.7	65.9	71.9	
Transplant year				0.5546
2005	16.2	13.3	16.3	
2006	15.8	16.9	15.8	
2007	14.5	15.3	14.5	
2008	13.0	14.5	13.0	
2009	13.8	14.9	13.7	
2010	13.6	10.4	13.7	
2011	13.0	14.9	13.0	
Cause of ESKD				0.0003
Cystic/hereditary	4.6	6.4	4.6	
Diabetes mellitus	18.5	8.8	18.9	
GN/vasculitis	41.8	51.0	41.5	
Hypertension/LVD	14.1	12.1	14.2	
Other	20.9	21.7	20.9	
Immunosuppression at time of transplant				
Cyclosporine/tacrolimus	92.8	96.0	92.7	0.0466
Mycophenolate	89.4	88.4	89.5	0.5698
Sirolimus	6.0	7.2	6.0	0.4175
Prednisone/steroids	92.4	91.2	92.5	0.4430
GFR at six months after transplantation (ml/min/1.73 m^2)^*	61.3 (23.3)	65.8 (23.2)	61.2 (23.3)	0.0021
				<0.0001
≥60	45.6	57.8	45.2	
<60	47.8	41.4	48.0	
Missing creatinine value	6.6	0.8	6.8	
Duration of dialysis (years)				0.0152
<3	48.3	55.8	48.0	
≥3	51.7	44.2	52.0	
Comorbidities				
Diabetes mellitus	17.7	10.0	18.0	0.0013
Hypertension	72.6	76.3	72.4	0.1782
Congestive heart failure	5.5	6.4	5.5	0.5328
Region				0.3679
Northeast	19.1	20.9	19.1	
South	39.0	33.3	39.2	
Midwest	23.2	23.7	23.1	
West	18.7	22.1	18.6	
Unknown	0.1	0.0	0.1	
Prior transplant recipient	17.8	18.5	17.8	0.7901
Died during follow-up	5.5	1.2	5.6	0.0027
Graft failure during follow-up	15.5	13.3	15.6	0.3163
Conception year				
Year 1		26.9		
Year 2		42.6		
Year 3		43.0		

*Reported in mean (standard deviation); ESKD, end stage kidney disease; GN, glomerulonephritis; LVD, large vessel disease; GFR, glomerular filtration rate

**Table 2**shows the characteristics of pregnant women by post-transplant year of conception. The mean age of pregnant women at transplant was 27±6 years. Women who conceived during the first year had a higher mean age at transplant than women who conceived during the second or third year. There was a non-significant change in race across the conception years: black women represented the largest group of conceptions within the first year post-transplant, and white and Hispanic women were the largest groups with conception third year post-transplant. Unadjusted pregnancy rates were higher in women in the second year (15.98 PTPY; 95% CI, 13.17–19.21) and third year post-transplant (16.93 PTPY; 95% CI, 13.96–20.36) than in the first year post-transplant (8.96 PTPY; 95% CI, 6.95–11.38).

**Table 2:** Baseline characteristics of pregnancies among women of childbearing age separated by conception year post-transplant.

Characteristics of Pregnancies	All^+^ (n = 293)	Conception within first year post-transplant (n = 67)	Conception within second year post-transplant (n = 113)	Conception within third year post-transplant (n = 113)	P Value
Age at transplantation (years)*	27 (6)	29 (7)	26 (6)	27 (6)	0.0088
	** **		** **		0.1414
15–19	11.6	7.5	13.3	12.4	
20–24	20.1	19.4	21.2	19.5	
25–29	30.0	23.9	31.9	31.9	
30–34	24.6	23.9	22.1	27.4	
35–45	13.7	25.4	11.5	8.9	
Race					0.1307
Black	29.4	40.3	31.9	20.4	
Hispanic	30.0	20.9	31.0	34.5	
White	35.2	32.8	32.7	38.9	
Other/unknown	5.5	6.0	4.4	6.2	
Donor type					0.7026
Living	34.5	34.3	31.9	37.2	
Deceased	65.5	65.7	68.1	62.8	
Cause of ESKD					0.6594
Cystic/hereditary	6.8	4.5	8.0	7.1	
Diabetes mellitus	8.2	10.5	7.1	8.0	
GN/vasculitis	51.2	58.2	46.9	51.3	
Hypertension/LVD	12.0	13.4	11.5	11.5	
Other	21.8	13.4	26.6	22.1	
GFR at six months after transplantation (ml/min/1.73 m^2)^*	66.0 (23.0)	63.6 (20.3)	65.2 (23.9)	68.1 (23.5)	0.3959
					0.8371
≥60	57.0	55.2	54.0	61.1	
<60	42.0	43.3	45.1	38.1	
Missing creatinine value	1.0	1.5	0.9	0.9	
Duration of dialysis (years)					0.4004
<3	54.9	47.8	56.6	57.5	
≥3	45.1	52.2	43.4	42.5	
Comorbidities					
Diabetes mellitus	9.2	13.4	5.3	10.6	0.1533
Hypertension	75.1	83.6	73.5	71.7	0.1785
Congestive heart failure	6.1	13.4	3.5	4.4	0.0176
Outcomes of pregnancy					
Live birth	39.4	36.8	38.9	41.2	
Still birth	3.1	2.9	1.8	4.4	
					
Ectopic	1.4	1.5	0.0	2.6	
Abortion	30.9	26.5	35.4	29.0	
Unknown	25.4	32.4	23.9	22.8	
Region					0.0905
Northeast	19.8	23.9	23.0	14.2	
South	32.1	31.3	35.4	29.2	
Midwest	23.9	29.9	19.5	24.8	
West	24.2	14.9	22.1	31.9	

*Reported in mean (standard deviation); ESKD, end stage renal disease; GN, glomerulonephritis; LVD, large vessel disease; GFR, glomerular filtration rate

^+^Multiple pregnancies are possible per woman

### Rates of pregnancy

**Fig 2:**
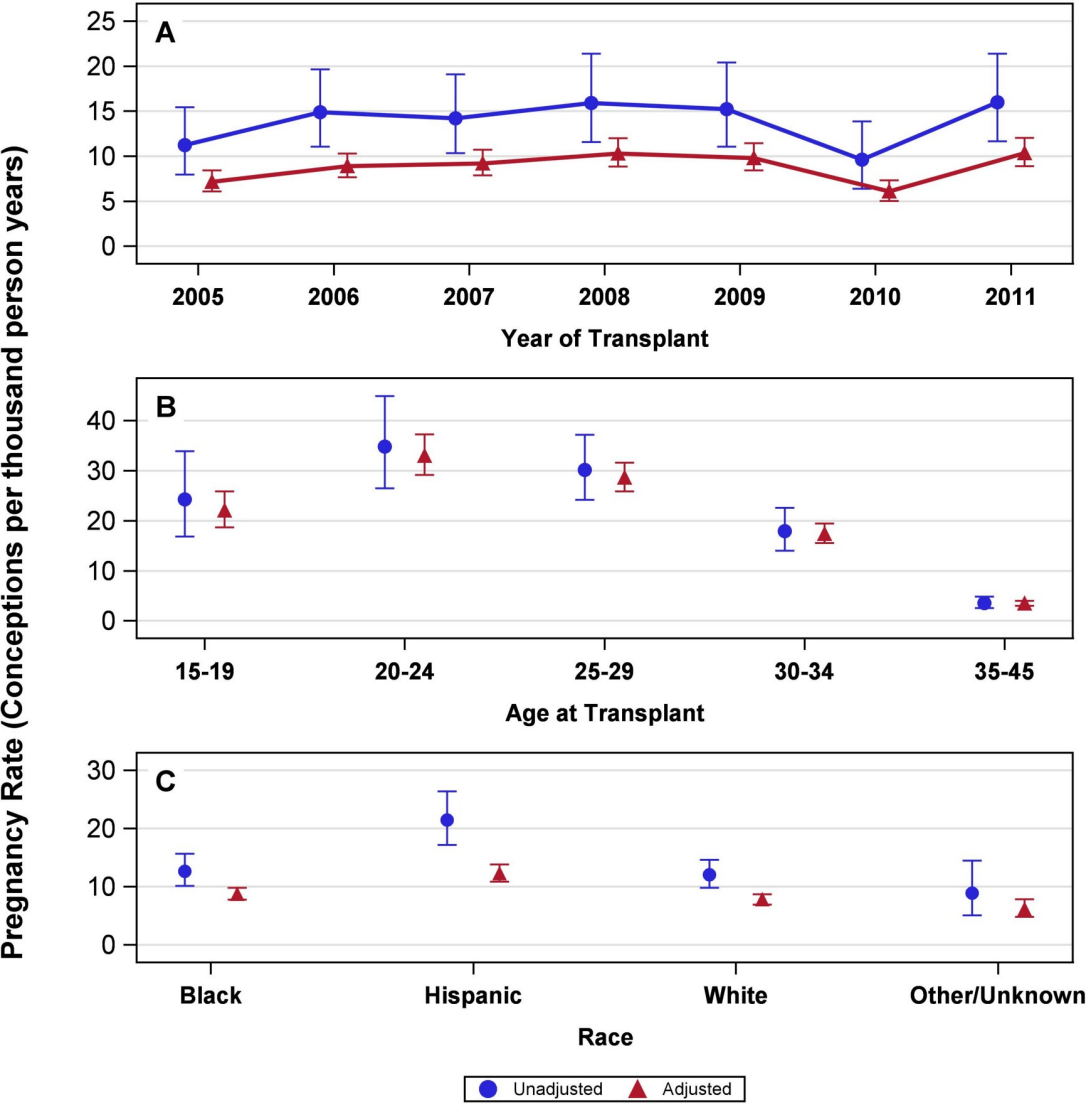
Adjusted and unadjusted rates of pregnancy in transplant recipient of child-bearing age (A) by year of transplant, (B) by age at transplant, and (C) by race. (A) The pregnancy rates by year of transplant, unadjusted and adjusted by age and race, and 95% confidence intervals; P value < 0.0001 for difference in rates across seven groups by year of transplant. 2005, 11.3 (8.0–15.4) and 7.2 (6.1–8.4); 2006, 14.9 (11.1–19.6) and 8.9 (7.7–10.3); 2007, 14.2 (10.3–19.1) and 9.2 (7.9–10.7); 2008, 15.9 (11.6–21.4) and 10.3 (8.8–12.0); 2009, 15.2 (11.1–20.4) and 9.8 (8.4–11.4); 2010, 9.6 (6.4–13.9) and 6.1 (5.0–7.3); 2011, 16.0 (11.7–21.4) and 10.4 (8.9–12.0). (B) The pregnancy rates by age at transplant, unadjusted and adjusted by race and year of transplant, and 95% confidence intervals; P value < 0.0001 for difference in rates between age groups. 15–19 years, 24.3 (16.8–33.9) and 22.0 (18.7–25.9); 20–24 years, 34.8 (26.5–44.9) and 33.0 (29.2–37.3); 25–29 years, 30.2 (24.2–37.2) and 28.6 (25.9–31.7); 30–34 years, 17.9 (14.0–22.6) and 17.4 (15.6–19.4); 35–45 years, 3.6 (2.6–4.9) and 3.5 (3.0–4.0). (C) The pregnancy rates by race, unadjusted and adjusted by age at transplant and year of transplant, and 95% confidence intervals; P value < 0.0001 for difference in rates across four races. Black, 12.7 (10.1–15.7) and 8.7 (7.8–9.8); Hispanic, 21.4 (17.2–26.4) and 12.3 (10.9–13.8); White, 12.1 (9.9–14.6) and 7.8 (7.0–8.7); Other/unknown, 8.9 (5.1–14.5) and 6.2 (4.9–7.8).

The majority of women (84.3%) had a single pregnancy, 14.1% of women had two pregnancies, and 1.6% of women had three or more pregnancies. Overall, the rate of pregnancy was 13.8 PTPY (95% CI, 12.3–15.5). Pregnancy rates were roughly constant for women transplanted in the years 2005–2011, with slightly lower rates in 2005 and 2010 **(Fig 2A).** The pregnancy rate adjusted for race and transplant year was highest in women aged 20–24 years at transplant (33.0 PTPY; 95% CI, 29.2–37.3) followed by 25–29 years (28.6 PTPY; 95% CI, 25.9–31.7), 15–19 years (22.0 PTPY; 95% CI, 18.7–25.9), 30–34 years (17.4 PTPY; 95% CI, 15.6–19.4), and 35–45 years (3.5 PTPY; 95% CI, 3.0–4.0) **(Fig 2B).** The pregnancy rate adjusted for age at transplant and transplant year was highest in Hispanic women (12.3 PTPY; 95% CI, 10.9–13.8) followed by blacks (8.7 PTPY; 95% CI, 7.8–9.8) and whites (7.8 PTPY; 95% CI, 7.0–8.7) **(Fig 2C).** Similar patterns were seen among unadjusted and adjusted rates. Sensitivity analyses were similar to the main analyses except in the rates by post-transplant year. In the first sensitivity analysis where we excluded pregnancies with less than 40 weeks of follow up, we found a slight increase in rates for the third year. In the second sensitivity analysis, where we removed women from the model when they turned 46 years old, the pregnancy rate in the second post transplant year was 2.4 PTPY higher, and the pregnancy rate in the third post transplant year was 3.7 PTPY higher **(S1 Table)**.

### Adjusted odds ratios for factors associated with pregnancies

**Fig 3:**
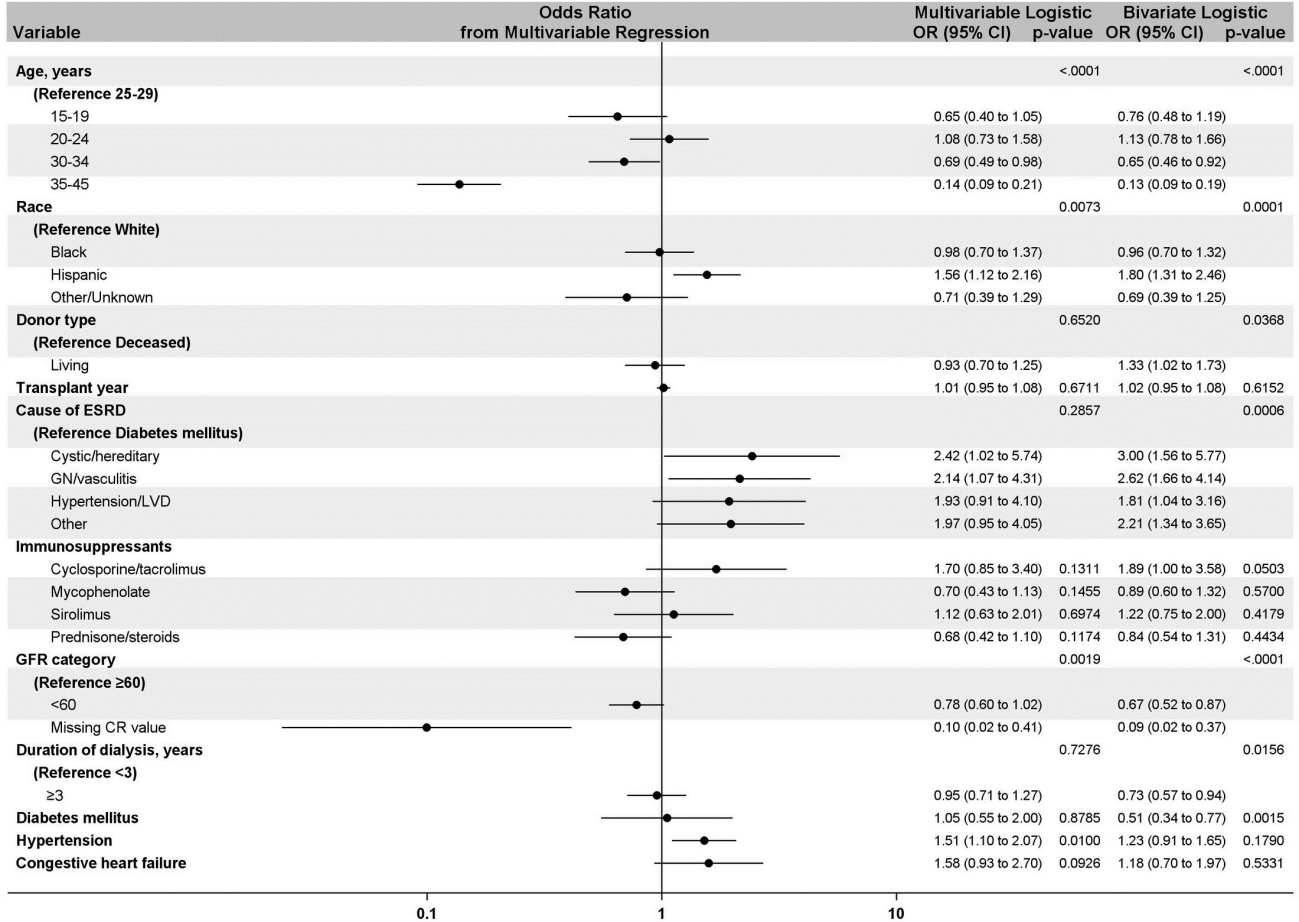
Main effects model showing factors associated with pregnancy in kidney transplant recipients in the bivariate and multivariable logistic analysis.

Compared to women aged 25–29 years at transplant, women aged 30–34 years (OR, 0.69; CI, 0.49–0.98) and 35–45 years (OR, 0.14; CI, 0.09–0.21) at transplant had a lower likelihood of becoming pregnant during the follow-up period. Hispanic women had a higher likelihood of pregnancy than did white women (OR, 1.56; CI, 1.12–2.16). Compared to women with ESKD due to diabetes, pregnancy was more likely in women with ESKD due to cystic disease (OR, 2.42; CI, 1.02–5.74) or glomerulonephritis (OR, 2.14; CI, 1.07–4.31). The comorbidity of hypertension was associated with a higher likelihood of pregnancy (OR, 1.51; CI, 1.10–2.07). Donor type, duration of dialysis, and the comorbidity of diabetes were significant in bivariate models but not in the multivariable model. Similarly, GFR < 60 six months post-transplantation was associated with lower odds of pregnancy in the bivariate model as compared to GFR ≥ 60, but was not significant in multivariable analyses. Types of immunosuppression at the time of transplant did not impact pregnancy rates in bivariate or multivariable analyses, and there was no trend in likelihood of pregnancy by transplant year **(Fig 3).** Sensitivity analyses produced results highly consistent to those from our main analyses **(S3 Table).**

Overall, fetal outcomes were known for only 191 of 293 pregnancies. The percentages of fetal outcomes were as follow: live births (39.4%, n = 116), still births (3.1%, n = 9), abortions (30.9%, n = 91), ectopic pregnancies (1.4%, n = 4), and unknown outcomes (25.4%, n = 75) (295 total birth outcomes due to two pregnancies which resulted in both live birth and still birth).

## Discussion

Using the USRDS, the current study determines the incidence of pregnancy in the first three years post-transplant from 2005–2011 and factors associated with pregnancy among Medicare-insured kidney transplant recipients of childbearing age in the United States. Our study shows that rates of pregnancy in kidney transplant recipients have not declined over time. Rates of pregnancy were highest in women aged 20–29 years at transplant and Hispanic women, and during the second and third year post kidney transplant. While type of donor, duration of dialysis, and type of immunosuppression did not impact likelihood of pregnancy, age, race, ESKD cause, and hypertension were significantly associated with pregnancy.

Encouragingly, we show for the first time that pregnancy rates in women with kidney transplants remained constant and did not show a decline, and in fact were higher than those reported from 1990–2003 (36.8 per 1000 women vs. 32.7 per 1000 women) [17]. In our study, the rates of pregnancy remained more or less constant from 2005–2011 except in 2005 and 2010. Lower pregnancy rates in 2010 could be related to lower pregnancy rates in the general population in 2010 [20]. We speculate that the pregnancy rates did not decline in the recent decade due to an increased awareness and information about risk factors, graft outcomes, interdisciplinary approaches, and established guidelines for immunosuppression management during pregnancy in women with kidney transplants [21, 22]. In the general population in the United States, the estimated pregnancy rate was 105.5 pregnancies per 1,000 women in 2008 [20]. Why do pregnancy rates in women with a kidney transplant remain lower than that of the general population? We believe that this could be related to the patient and physician related factors. It’s possible that physicians do not encourage pregnancy due to the risk of adverse maternal and fetal outcomes, or patients are skeptical due to a fear of deterioration of kidney allograft. The overall rates may also be influenced by the skew towards older women in the transplant population, so that about half are in the oldest age group, which had the lowest individual rate of pregnancy. The associated risk of teratogenicity with use of immunosuppression could be another factor [23]. Information regarding the safety of immunosuppressant drugs in pregnancy accumulates slowly and is hampered by the absence of comprehensive post marketing ascertainment of pregnancy outcomes in women treated with immunosuppressant medications. For example, belatacept is available but its association and safety with pregnancy is known only from case reports [24]. Infertility due to chronic illness and patients choosing to use effective birth control options could be other contributing factors to lower rates of pregnancy in kidney transplant recipients as compared to general population.

The pregnancy rates in the first post-transplant year were lower than the second and third post-transplant year. Although this pattern was consistent with prior literature, our overall rates were higher than what has been reported [17]. Pregnancy rates are expected to be lower in the first year post transplant, since allograft function may take up to six months to stabilize. Additionally, usually by one year the immunosuppression is lowered and the post-transplant prophylaxis is completed [3]. Since the appropriate time of conception after kidney transplant remains an area of disagreement, with the current recommendations varying between one to three years post-transplant, this could be another reason for the lower pregnancy rates in the first year post-transplant [21, 22].

Our study shows that Hispanic women had the highest pregnancy rates amongst all racial groups, with 56% higher odds of pregnancy in kidney transplant recipients compared to whites, while black women were not statistically different from white women. This is in contrast to what has been reported about the general population, in which black women (144.3 per 1,000) had the highest rates of pregnancies followed by Hispanic women (136.9), and were about 60 percent higher than the rate for non-Hispanic white women (87.5) [20]. A UK-based study by Braham et al did not find a significant difference in pregnancy rates by race, but compared only white vs. non-white [25]. Greater family support systems, cultural characteristics that influence individual health and lifestyle behaviors, genetic factors, and social networks in the Hispanic population may play a contributing role in the higher rates of pregnancy in kidney transplant recipients [26, 27]. Socioeconomic status and health literacy level could be other factors that may explain differences in the rates of pregnancy among racial groups, but these patient level variables are not captured in the USRDS database and remain a limitation of the study.

Additionally, ESKD caused by cystic disease, or glomerulonephritis was associated with higher odds of pregnancy as compared to diabetes. While reasons remain unclear, we speculate that this may be related to women being overall healthier than those having ESKD due to diabetes. Our study also showed that hypertension as a comorbidity was associated with a higher likelihood of pregnancy in kidney transplant recipients. This could be simply because hypertension is commonly associated with polycystic kidney disease and glomerulonephritis, or because cystic disease and glomerulonephritis was prominently represented by the group of women who became pregnant post kidney transplant [28]. Although elevated creatinine is associated with infertility and lower likelihood of pregnancy in women with chronic kidney disease, kidney function was not associated with likelihood of pregnancy in our study, which could be due to differing proportions of missing values for serum creatinine among women who became pregnant and those who did not [29, 30]. Also to be noted, the mean GFR was > 60 in the study cohort, and was measured at six months after transplantation. It’s possible that the kidney function improved further close to their conception time. When we measured GFR within one year of conception, there were again large numbers of missing values for serum creatinine. Since elevated pre-pregnancy creatinine ≥ 1.4 mg/dl is a well-known risk factor for poor pregnancy outcomes in kidney transplant recipients, patients should be counseled for this while contemplating pregnancy [3, 7].

The live birth rate in our study was 39%, much lower than reported in other studies including those from National Transplant Pregnancy Registry (NTPR) (71–76%), and the US general population (62%) [8, 13, 31, 32]. Most likely this higher live birth rate reflects a selection bias due to healthier women getting pregnant in addition to NTPR being a voluntary registry in which only successful pregnancy outcomes may be reported. Another study from the US reported a lower live birth rate of 55% in kidney transplant recipients due to underreporting of fetal loss [17]. In the present study, overall outcomes were unknown in 25% of the pregnancies in our study. We therefore anticipate the live birth rates to be much higher, and closer to the rates of general population. Additionally, the stillbirth rate was higher than in the United States general population (3.1% vs. 0.6%), and could be associated with the use of immunosuppression, maternal risk factors of drug treated hypertension, preeclampsia and allograft dysfunction and higher likelihood of adverse fetal outcomes of preterm deliveries, small for gestation age babies and low birth weight babies among kidney transplant recipients [33–36].

Our study has several limitations. First, the study provides information regarding pregnancy only during the first three post-transplant years while patients maintained Medicare insurance coverage. However, by including patients with complete Medicare coverage, we maximized the chance of capturing events, thus avoiding the potential shortfalls of registries dependent on voluntary reporting or patient recall. Second, information regarding the use of immunosuppressive medications was available at the time of transplantation and not at the time of conception. Third, although we adapted a prior validated method based on pregnancy-related claims to identify episodes of pregnancy, about 20% of the pregnancies were identified based only on codes at the end of pregnancy; therefore, pregnancies in the third post-transplant year may be under-reported as the end did not happen during our follow-up period. However, sensitivity analyses found that that our conclusions were similar when limiting conception dates, except that the third post-transplant year rates were slightly higher. Fourth, information on socioeconomic status and health literacy, factors that can impact pregnancy, were not available for our analysis. Importantly, the strength of the current study is that it is one of the largest studies in US using an administrative database that provides information about the factors associated with pregnancy and pregnancy rates among kidney transplant recipients.

## Conclusion

In conclusion, the pregnancy rate after kidney transplantation has remained roughly constant during the recent decade. Pregnancy rates were higher in the second and third year post-transplant year than the first post-transplant year. Age, Hispanic race, and cause of ESKD are significant factors associated with the likelihood of pregnancy. In kidney transplant recipients, the still birth rate is higher as compared to general population. These findings have shared decision-making implications for both kidney transplant patients and their physicians.

## Supporting information

S1 Table Discharge diagnoses and medical procedures indicative of occurrence of pregnancy and pregnancy related events. (DOCX)

S2 Table Pregnancy rates with sensitivity analysis performed with conception dates cut off 40 weeks before the end of follow up and removal of women older than 45 years. (DOCX)

S3 Table Pregnancy rates with sensitivity analyses performed with removal of deaths and graft failure, and removal of those with missing creatinine. (DOCX)

## References

[1] Piccoli GB, Attini R, Vasario E, Conijn A, Biolcati M, D'Amico F, et al Pregnancy and chronic kidney disease: a challenge in all CKD stages. Clinical journal of the American Society of Nephrology: CJASN. 2010;5(5):844–55. Epub 2010/04/24. 10.2215/CJN.07911109 20413442 PMC2863984

[2] Palmer BF. Sexual dysfunction in men and women with chronic kidney disease and end-stage kidney disease. Advances in renal replacement therapy. 2003;10(1):48–60. Epub 2003/03/05. 10.1053/jarr.2003.50003 . 12616463

[3] Shah S, Verma P. Overview of Pregnancy in Renal Transplant Patients. International journal of nephrology. 2016;2016:4539342 Epub 2017/01/04. 10.1155/2016/4539342 28042483 PMC5155089

[4] Saha MT, Saha HH, Niskanen LK, Salmela KT, Pasternack AI. Time course of serum prolactin and sex hormones following successful renal transplantation. Nephron. 2002;92(3):735–7. Epub 2002/10/10. doi: 64079. 10.1159/000064079 . 12372970

[5] Davison JM. The effect of pregnancy on kidney function in renal allograft recipients. Kidney Int. 1985;27(1):74–9. Epub 1985/01/01. 10.1038/ki.1985.12 . 3884881

[6] Keitel E, Bruno RM, Duarte M, Santos AF, Bittar AE, Bianco PD, et al Pregnancy outcome after renal transplantation. Transplant Proc. 2004;36(4):870–1. Epub 2004/06/15. 10.1016/j.transproceed.2004.03.089 . 15194297

[7] Stratta P, Canavese C, Giacchino F, Mesiano P, Quaglia M, Rossetti M. Pregnancy in kidney transplantation: satisfactory outcomes and harsh realities. Journal of nephrology. 2003;16(6):792–806. Epub 2004/01/23. PubMed . 14736006

[8] Shah S, Venkatesan RL, Gupta A, Sanghavi MK, Welge J, Johansen R, et al Pregnancy outcomes in women with kidney transplant: Metaanalysis and systematic review. BMC nephrology. 2019;20(1):24 Epub 2019/01/25. 10.1186/s12882-019-1213-5 30674290 PMC6345071

[9] McKay DB, Josephson MA. Pregnancy after kidney transplantation. Clinical journal of the American Society of Nephrology: CJASN. 2008;3 Suppl 2:S117–25. Epub 2008/03/20. 10.2215/cjn.02980707 18308999 PMC3152271

[10] McKay DB, Josephson MA. Pregnancy in recipients of solid organs—effects on mother and child. N Engl J Med. 2006;354(12):1281–93. Epub 2006/03/24. 10.1056/NEJMra050431 . 16554530

[11] Davison JM, Redman CW. Pregnancy post-transplant: the establishment of a UK registry. British journal of obstetrics and gynaecology. 1997;104(10):1106–7. Epub 1997/10/23. 10.1111/j.1471-0528.1997.tb10930.x . 9332984

[12] Rizzoni G, Ehrich JH, Broyer M, Brunner FP, Brynger H, Fassbinder W, et al Successful pregnancies in women on renal replacement therapy: report from the EDTA Registry. Nephrol Dial Transplant. 1992;7(4):279–87. Epub 1992/01/01. 10.1093/oxfordjournals.ndt.a092129 . 1317516

[13] Coscia LA, Constantinescu S, Moritz MJ, Frank AM, Ramirez CB, Maley WR, et al Report from the National Transplantation Pregnancy Registry (NTPR): outcomes of pregnancy after transplantation. Clinical transplants. 2010:65–85. Epub 2010/01/01. PubMed . 21698831

[14] Levidiotis V, Chang S, McDonald S. Pregnancy and maternal outcomes among kidney transplant recipients. Journal of the American Society of Nephrology: JASN. 2009;20(11):2433–40. Epub 2009/10/03. 10.1681/ASN.2008121241 19797167 PMC2799176

[15] Hornbrook MC, Whitlock EP, Berg CJ, Callaghan WM, Bachman DJ, Gold R, et al Development of an algorithm to identify pregnancy episodes in an integrated health care delivery system. Health services research. 2007;42(2):908–27. Epub 2007/03/17. 10.1111/j.1475-6773.2006.00635.x 17362224 PMC1955367

[16] Blotiere PO, Weill A, Dalichampt M, Billionnet C, Mezzarobba M, Raguideau F, et al Development of an algorithm to identify pregnancy episodes and related outcomes in health care claims databases: An application to antiepileptic drug use in 4.9 million pregnant women in France. Pharmacoepidemiology and drug safety. 2018;27(7):763–70. Epub 2018/05/16. 10.1002/pds.4556 29763992 PMC6055607

[17] Gill JS, Zalunardo N, Rose C, Tonelli M. The pregnancy rate and live birth rate in kidney transplant recipients. Am J Transplant. 2009;9(7):1541–9. Epub 2009/05/23. 10.1111/j.1600-6143.2009.02662.x . 19459800

[18] Kshirsagar AV, Manickam RN, Mu Y, Flythe JE, Chin AI, Bang H. Area-level poverty, race/ethnicity & dialysis star ratings. PloS one. 2017;12(10):e0186651 Epub 2017/10/19. 10.1371/journal.pone.0186651 29040342 PMC5645143

[19] U.S. Renal Data System. 2015 Researcher’s Guide to the USRDS Database. National Institutes of Health, National Institute of Diabetes and Digestive and Kidney Diseases, Bethesda, MD 2015.

[20] Ventura SJ, Curtin SC, Abma JC, Henshaw SK. Estimated pregnancy rates and rates of pregnancy outcomes for the United States, 1990–2008. National vital statistics reports: from the Centers for Disease Control and Prevention, National Center for Health Statistics, National Vital Statistics System. 2012;60(7):1–21. Epub 2012/09/14. PubMed . 22970648

[21] European best practice guidelines for renal transplantation. Section IV: Long-term management of the transplant recipient. IV.10. Pregnancy in renal transplant recipients. Nephrol Dial Transplant. 2002;17 Suppl 4:50–5. Epub 2002/07/02. PubMed . 12091650

[22] McKay DB, Josephson MA, Armenti VT, August P, Coscia LA, Davis CL, et al Reproduction and transplantation: report on the AST Consensus Conference on Reproductive Issues and Transplantation. Am J Transplant. 2005;5(7):1592–9. Epub 2005/06/10. 10.1111/j.1600-6143.2005.00969.x . 15943616

[23] Coscia LA, Constantinescu S, Davison JM, Moritz MJ, Armenti VT. Immunosuppressive drugs and fetal outcome. Best practice & research Clinical obstetrics & gynaecology. 2014;28(8):1174–87. Epub 2014/09/02. 10.1016/j.bpobgyn.2014.07.020 . 25175414

[24] Combs J, Kagan A, Boelkins M, Coscia L, Moritz M, Hofmann RM. Belatacept during pregnancy in renal transplant recipients: Two case reports. Am J Transplant. 2018;18(8):2079–82. Epub 2018/05/03. 10.1111/ajt.14911 . 29719109

[25] Bramham K, Nelson-Piercy C, Gao H, Pierce M, Bush N, Spark P, et al Pregnancy in renal transplant recipients: a UK national cohort study. Clinical journal of the American Society of Nephrology: CJASN. 2013;8(2):290–8. Epub 2012/10/23. 10.2215/CJN.06170612 23085724 PMC3562860

[26] Liao Y, Cooper RS, Cao G, Durazo-Arvizu R, Kaufman JS, Luke A, et al Mortality patterns among adult Hispanics: findings from the NHIS, 1986 to 1990. American journal of public health. 1998;88(2):227–32. Epub 1998/03/10. 10.2105/ajph.88.2.227 9491012 PMC1508177

[27] Abraido-Lanza AF, Dohrenwend BP, Ng-Mak DS, Turner JB. The Latino mortality paradox: a test of the "salmon bias" and healthy migrant hypotheses. American journal of public health. 1999;89(10):1543–8. Epub 1999/10/08. 10.2105/ajph.89.10.1543 10511837 PMC1508801

[28] Chapman AB, Stepniakowski K, Rahbari-Oskoui F. Hypertension in autosomal dominant polycystic kidney disease. Advances in chronic kidney disease. 2010;17(2):153–63. Epub 2010/03/12. 10.1053/j.ackd.2010.01.001 20219618 PMC2845913

[29] Holley JL, Schmidt RJ. Changes in fertility and hormone replacement therapy in kidney disease. Advances in chronic kidney disease. 2013;20(3):240–5. Epub 2013/08/10. 10.1053/j.ackd.2013.01.003 . 23928388

[30] Orofino L, Quereda C, Lamas S, Orte L, Gonzalo A, Mampaso F, et al Hypertension in primary chronic glomerulonephritis: analysis of 288 biopsied patients. Nephron. 1987;45(1):22–6. Epub 1987/01/01. 10.1159/000184065 . 3808144

[31] Martin J. Births in the United States, 2016 NCHS data brief, no 287. Hyattsville, MD: National Center for Health Statistics: 2017.

[32] Deshpande NA, James NT, Kucirka LM, Boyarsky BJ, Garonzik-Wang JM, Montgomery RA, et al Pregnancy outcomes in kidney transplant recipients: a systematic review and meta-analysis. Am J Transplant. 2011;11(11):2388–404. Epub 2011/07/29. 10.1111/j.1600-6143.2011.03656.x . 21794084

[33] MacDorman M. Fetal and Perinatal Mortality: United States, 2013. National Vital Statistics Reports; vol 64 no 8. Hyattsville, MD: National Center for Health Statistics 2015. 2015. 26222771

[34] Cruz Lemini MC, Ibarguengoitia Ochoa F, Villanueva Gonzalez MA. Perinatal outcome following renal transplantation. International journal of gynaecology and obstetrics: the official organ of the International Federation of Gynaecology and Obstetrics. 2007;96(2):76–9. Epub 2007/01/24. 10.1016/j.ijgo.2006.11.006 . 17239381

[35] Little MA, Abraham KA, Kavanagh J, Connolly G, Byrne P, Walshe JJ. Pregnancy in Irish renal transplant recipients in the cyclosporine era. Irish journal of medical science. 2000;169(1):19–21. Epub 2000/06/10. PubMed . 10846851 10.1007/BF03170477

[36] Majak GB, Reisaeter AV, Zucknick M, Lorentzen B, Vangen S, Henriksen T, et al Preeclampsia in kidney transplanted women; Outcomes and a simple prognostic risk score system. PloS one. 2017;12(3):e0173420 Epub 2017/03/21. 10.1371/journal.pone.0173420 28319175 PMC5358770

