# Supplementary material for: Racial disparities and factors associated with pregnancy in kidney transplant recipients in the United States
Source: PLoS One. 2019 Aug 9;14(8):e0220916. doi: 10.1371/journal.pone.0220916 (PMC6688836; doi:10.1371/journal.pone.0220916)
Supplement: S1 Table — (DOCX) [file pone.0220916.s001.docx]

S1 Table. Discharge diagnoses and medical procedures indicative of occurrence of pregnancy and pregnancy related events

**Live Birth**

ICD-9 diagnoses: 641.01, 641.11, 641.21, 641.31, 641.81, 641.91, 642.01, 642.02, 642.11, 642.12, 642.21, 642.22, 642.31, 642.32, 642.41, 642.42, 642.51, 642.52, 642.61, 642.62, 642.71, 642.72, 642.91, 642.92, 643.01, 643.11, 643.21, 643.81, 643.91, 644.2, 645.01, 645.11, 645.21, 646.11, 646.12, 646.21, 646.22, 646.31, 646.41, 646.42, 646.51, 646.52, 646.61, 646.62, 646.71, 646.81, 646.82, 646.91, 647.01, 647.02, 647.11, 647.12, 647.21, 647.22, 647.31, 647.32, 647.41, 647.42, 647.51, 647.52, 647.61, 647.62, 647.81, 647.82, 647.91, 647.92, 648.01, 648.02, 648.11, 648.12, 648.21, 648.22, 648.31, 648.32, 648.41, 648.42, 648.51, 648.52, 648.61, 648.62, 648.71, 648.72, 648.82, 648.91, 648.92, 650, 651.01, 651.11, 651.21, 651.31, 651.41, 651.51, 651.61, 651.81, 651.91, 652.01, 652.11, 652.21, 652.31, 652.41, 652.51, 652.61, 652.71, 652.81, 652.91, 653.01, 653.11, 653.21, 653.31, 653.41, 653.51, 653.61, 653.71, 653.81, 653.91, 654.01, 654.02, 654.11, 654.12, 654.21, 654.31, 654.32, 654.41, 654.42, 654.51, 654.52, 654.61, 654.62, 654.71, 654.72, 654.81, 654.82, 654.91, 654.92, 655.01, 655.11, 655.21, 655.31, 655.41, 655.51, 655.61, 655.71, 655.81, 655.91, 656.01, 656.11, 656.21, 656.31, 656.51, 656.61, 656.71, 656.81, 656.91, 657.01, 658.01, 658.11, 658.21, 658.3, 658.30, 658.31, 658.33, 658.41, 658.81, 658.91, 659.0, 659.00, 659.01, 659.03, 659.1, 659.10, 659.11, 659.13, 659.2, 659.20, 659.21, 659.23, 659.3, 659.30, 659.31, 659.33, 659.41, 659.51, 659.61, 659.71, 659.8, 659.80, 659.81, 659.83, 659.9, 659.90, 659.91, 659.93, 660, 660.0, 660.00, 660.01, 660.03, 660.1, 660.10, 660.11, 660.13, 660.2, 660.20, 660.21, 660.23, 660.3, 660.30, 660.31, 660.33, 660.4, 660.40, 660.41, 660.43, 660.5, 660.50, 660.51, 660.53, 660.6, 660.60, 660.61, 660.63, 660.7, 660.70, 660.71, 660.73, 660.8, 660.80, 660.81, 660.83, 660.9, 660.90, 660.91, 660.93, 661, 661.0, 661.00, 661.01, 661.03, 661.1, 661.10, 661.11, 661.13, 661.2, 661.20, 661.21, 661.23, 661.3, 661.30, 661.31, 661.33, 661.4, 661.40, 661.41, 661.43, 661.9, 661.90, 661.91, 661.93, 662, 662.0, 662.00, 662.01, 662.03, 662.1, 662.10, 662.11, 662.13, 662.2, 662.20, 662.21, 662.23, 662.3, 662.30, 662.31, 662.33, 663, 663.0, 663.00, 663.01, 663.03, 663.1, 663.10, 663.11, 663.13, 663.2, 663.20, 663.21, 663.23, 663.3, 663.30, 663.31, 663.33, 663.4, 663.40, 663.41, 663.43, 663.5, 663.50, 663.51, 663.53, 663.6, 663.60, 663.61, 663.63, 663.8, 663.80, 663.81, 663.83, 663.9, 663.90, 663.91, 663.93, 664, 664.0, 664.00, 664.01, 664.1, 664.10, 664.11, 664.2, 664.20, 664.21, 664.3, 664.30, 664.31, 664.4, 664.40, 664.41, 664.5, 664.50, 664.51, 664.8, 664.80, 664.81, 664.9, 664.90, 664.91, 665, 665.0, 665.00, 665.01, 665.03, 665.1, 665.10, 665.11, 665.2, 665.20, 665.22, 665.3, 665.30, 665.31, 665.4, 665.40, 665.41, 665.5, 665.50, 665.51, 665.6, 665.60, 665.61, 665.7, 665.70, 665.71, 665.72, 665.8, 665.80, 665.81, 665.82, 665.83, 665.9, 665.90, 665.91, 665.92, 665.93, 666, 666.0, 666.00, 666.02, 666.1, 666.10, 666.12, 667, 667.0, 667.00, 667.02, 667.1, 667.10, 667.12, 668, 668.0, 668.00, 668.01, 668.02, 668.03, 668.1, 668.10, 668.11, 668.12, 668.13, 668.2, 668.20, 668.21, 668.22, 668.23, 668.8, 668.80, 668.81, 668.82, 668.83, 668.9, 668.90, 668.91, 668.92, 668.93, 669, 669.0, 669.00, 669.01, 669.02, 669.03, 669.1, 669.10, 669.11, 669.12, 669.13, 669.2, 669.20, 669.21, 669.22, 669.23, 669.3, 669.30, 669.32, 669.4, 669.40, 669.41, 669.42, 669.43, 669.5, 669.50, 669.51, 669.6, 669.60, 669.61, 669.7, 669.70, 669.71, 669.8, 669.80, 669.81, 669.82, 669.83, 669.9, 669.90, 669.91, 669.92, 669.93, 673.01, 673.02, 673.11, 673.12, 673.21, 673.22, 673.31, 673.32, 673.81, 673.82, V24.0, V27, V27.0, V27.2, V27.3, V27.5, V27.6, V27.9, V30, V30.0, V30.00, V30.01, V30.1, V30.2, V31, V31.0, V31.00, V31.01, V31.1, V31.2, V32, V32.0, V32.00, V32.01, V32.1, V32.2, V33, V33.0, V33.00, V33.01, V33.1, V33.2, V34, V34.0, V34.00, V34.01, V34.1, V34.2, V35, V35.0, V35.00, V35.01, V35.1, V35.2, V36, V36.0, V36.00, V36.01, V36.1, V36.2, V37, V37.0, V37.00, V37.01, V37.1, V37.2, V39, V39.0, V39.00, V39.01, V39.1, V39.2

ICD-9 procedures: 72, 72.0, 72.1, 72.2, 72.2x, 72.3, 72.3x, 72.4, 72.5, 72.5x, 72.6, 72.7, 72.7x, 72.8, 72.9, 73.0, 73.0x, 73.1, 73.22, 73.3, 73.4, 73.5, 73.5x, 73.6, 73.8, 73.9, 73.9x, 74, 74.0‐2, 74.4, 74.9, 74.99, 75.4, 75.5, 75.5x, 75.6, 75.6x, 75.8

CPT codes: 00850, 00857, 00946, 00955, 01960-3, 01967-9, 59414, 59510, 59514, 59610, 59612, 59618, 59620

DRG codes, v. 24^*^: 370-377

DRG codes, v. 25^*^: 765-9, 774-6

**Still Birth**

ICD-9 diagnoses: 646.01, 656.41, V27.1, V27.4, V27.7

**Ectopic Pregnancies/ Trophoblastic Disease**

ICD-9 procedures: 66.62, 69.11, 74.3

CPT codes: 59120-1, 59130, 59135-6, 59140, 59150-1, 59870

DRG codes, v. 24^*^: 378

DRG codes, v. 25^*^: 777

**Abortion**

ICD-9 diagnoses: 631, 632, 634, 634.0, 634.00, 634.01, 634.02, 634.1, 634.10, 634.11, 634.12, 634.2, 634.20, 634.21, 634.22, 634.3, 634.30, 634.31, 634.32, 634.4, 634.40, 634.41, 634.42, 634.5, 634.50, 634.51, 634.52, 634.6, 634.60, 634.61, 634.62, 634.7, 634.70, 634.71, 634.72, 634.8, 634.80, 634.81, 634.82, 634.9, 634.90, 634.91, 634.92, 635, 635.0, 635.00, 635.01, 635.02, 635.1, 635.10, 635.11, 635.12, 635.2, 635.20, 635.21, 635.22, 635.3, 635.30, 635.31, 635.32, 635.4, 635.40, 635.41, 635.42, 635.5, 635.50, 635.51, 635.52, 635.6, 635.60, 635.61, 635.62, 635.7, 635.70, 635.71, 635.72, 635.8, 635.80, 635.81, 635.82, 635.9, 635.90, 635.91, 635.92, 636, 636.0, 636.00, 636.01, 636.02, 636.1, 636.10, 636.11, 636.12, 636.2, 636.20, 636.21, 636.22, 636.3, 636.30, 636.31, 636.32, 636.4, 636.40, 636.41, 636.42, 636.5, 636.50, 636.51, 636.52, 636.6, 636.60, 636.61, 636.62, 636.7, 636.70, 636.71, 636.72, 636.8, 636.80, 636.81, 636.82, 636.9, 636.90, 636.91, 636.92, 637, 637.0, 637.00, 637.01, 637.02, 637.1, 637.10, 637.11, 637.12, 637.2, 637.20, 637.21, 637.22, 637.3, 637.30, 637.31, 637.32, 637.4, 637.40, 637.41, 637.42, 637.5, 637.50, 637.51, 637.52, 637.6, 637.60, 637.61, 637.62, 637.7, 637.70, 637.71, 637.72, 637.8, 637.80, 637.81, 637.82, 637.9, 637.90, 637.91, 637.92, 640.81, 640.91

ICD-9 procedures: 69.01, 69.51, 74.91, 75.0

CPT codes: 01964, 59100, 59812, 59820-1, 59830, 59840-1, 59850-2, 59855-7

DRG codes, v. 24^*^: 380, 381

DRG codes, v. 25^*^: 770, 779

**Unknown Outcome**

ICD-9 diagnoses: 630, 633, 633.0, 633.1, 633.2, 633.8, 633.9, 638, 638.0, 638.1, 638.2, 638.3, 638.4, 638.5, 638.6, 638.7, 638.8, 638.9, 640, 640.0, 640.00, 640.01, 640.03, 640.8, 640.80, 640.83, 640.9, 640.90, 640.93, 641, 641.0, 641.00, 641.03, 641.1, 641.10, 641.13, 641.2, 641.20, 641.23, 641.3, 641.30, 641.33, 641.8, 641.80, 641.83, 641.9, 641.90, 641.93, 642, 642.0, 642.00, 642.03, 642.1, 642.10, 642.13, 642.2, 642.20, 642.23, 642.3, 642.30, 642.33, 642.4, 642.40, 642.43, 642.5, 642.50, 642.53, 642.6, 642.60, 642.63, 642.7, 642.70, 642.73, 642.9, 642.90, 642.93, 643, 643.0, 643.00, 643.03, 643.1, 643.10, 643.13, 643.2, 643.20, 643.23, 643.8, 643.80, 643.83, 643.9, 643.90, 643.93, 644, 644.0, 644.00, 644.03, 644.1, 644.10, 644.13, 644.20, 644.21, 645, 645.00, 645.03, 645.10, 645.13, 645.20, 645.23, 646, 646.0, 646.00, 646.03, 646.1, 646.10, 646.13, 646.2, 646.20, 646.23, 646.3, 646.30, 646.33, 646.4, 646.40, 646.43, 646.5, 646.50, 646.53, 646.6, 646.60, 646.63, 646.7, 646.70, 646.73, 646.8, 646.80, 646.83, 646.9, 646.90, 646.93, 647, 647.0, 647.00, 647.03, 647.1, 647.10, 647.13, 647.2, 647.20, 647.23, 647.3, 647.30, 647.33, 647.4, 647.40, 647.43, 647.5, 647.50, 647.53, 647.6, 647.60, 647.63, 647.8, 647.80, 647.83, 647.9, 647.90, 647.93, 648, 648.0, 648.00, 648.03, 648.1, 648.10, 648.13, 648.2, 648.20, 648.23, 648.3, 648.30, 648.33, 648.4, 648.40, 648.43, 648.44, 648.5, 648.50, 648.53, 648.6, 648.60, 648.63, 648.7, 648.70, 648.73, 648.8, 648.80, 648.81, 648.83, 648.9, 648.90, 648.93, 648.94, 651, 651.0, 651.00, 651.03, 651.1, 651.10, 651.13, 651.2, 651.20, 651.23, 651.3, 651.30, 651.33, 651.4, 651.40, 651.43, 651.5, 651.50, 651.53, 651.6, 651.60, 651.63, 651.8, 651.80, 651.83, 651.9, 651.90, 651.93, 652, 652.0, 652.00, 652.03, 652.1, 652.10, 652.13, 652.2, 652.20, 652.23, 652.3, 652.30, 652.33, 652.4, 652.40, 652.43, 652.5, 652.50, 652.53, 652.6, 652.60, 652.63, 652.7, 652.70, 652.73, 652.8, 652.80, 652.83, 652.9, 652.90, 652.93, 653, 653.0, 653.00, 653.03, 653.1, 653.10, 653.13, 653.2, 653.20, 653.23, 653.3, 653.30, 653.33, 653.4, 653.40, 653.43, 653.5, 653.50, 653.53, 653.6, 653.60, 653.63, 653.7, 653.70, 653.73, 653.8, 653.80, 653.83, 653.9, 653.90, 653.93, 654, 654.0, 654.00, 654.03, 654.1, 654.10, 654.13, 654.2, 654.20, 654.23, 654.3, 654.30, 654.33, 654.4, 654.40, 654.43, 654.5, 654.50, 654.53, 654.6, 654.60, 654.63, 654.7, 654.70, 654.73, 654.8, 654.80, 654.83, 654.9, 654.90, 654.93, 655, 655.0, 655.00, 655.03, 655.1, 655.10, 655.13, 655.2, 655.20, 655.23, 655.3, 655.30, 655.33, 655.4, 655.40, 655.43, 655.5, 655.50, 655.53, 655.6, 655.60, 655.63, 655.70, 655.73, 655.8, 655.80, 655.83, 655.9, 655.90, 655.93, 656, 656.0, 656.00, 656.03, 656.1, 656.10, 656.13, 656.2, 656.20, 656.23, 656.3, 656.30, 656.33, 656.4, 656.40, 656.43, 656.5, 656.50, 656.53, 656.6, 656.60, 656.63, 656.7, 656.70, 656.73, 656.8, 656.80, 656.83, 656.9, 656.90, 656.93, 657, 657.00, 657.03, 658, 658.0, 658.00, 658.03, 658.1, 658.10, 658.13, 658.2, 658.20, 658.23, 658.4, 658.40, 658.43, 658.8, 658.80, 658.83, 658.9, 658.90, 658.93, 659, 659.4, 659.40, 659.43, 659.5, 659.50, 659.53, 659.6, 659.60, 659.63, 659.7, 659.70, 659.73, 671, 671.0, 671.00, 671.03, 671.1, 671.10, 671.13, 671.2, 671.20, 671.23, 671.3, 671.30, 671.33, 671.40, 671.5, 671.50, 671.53, 671.8, 671.80, 671.83, 671.9, 671.90, 671.93, 673, 673.0, 673.00, 673.03, 673.1, 673.10, 673.13, 673.2, 673.20, 673.23, 673.3, 673.30, 673.33, 673.8, 673.80, 673.83, 674, 674.0, 674.00, 674.03, 674.14, 674.24, 674.4, 674.40, 674.8, 674.80, 674.9, 674.90, 675, 675.0, 675.00, 675.03, 675.1, 675.10, 675.13, 675.2, 675.20, 675.23, 675.8, 675.80, 675.83, 675.9, 675.90, 675.93, 676, 676.0, 676.00, 676.03, 676.1, 676.10, 676.13, 676.2, 676.20, 676.22, 676.23, 676.3, 676.30, 676.33, 676.4, 676.40, 676.43, 676.5, 676.50, 676.53, 676.6, 676.60, 676.63, 676.8, 676.80, 676.81, 676.82, 676.83, 676.84, 676.9, 676.90, 676.91, 676.92, 676.93, 676.94, 677, 768.0, 768.1, 792.3, 796.5, V22, V22.0, V22.1, V22.2, V23, V23.0, V23.1, V23.2, V23.3, V23.4, V23.5, V23.7, V23.8, V23.81, V23.82, V23.83, V23.84, V23.89, V23.9, V28, V28.0, V28.1, V28.2, V28.3, V28.4, V28.5, V28.6, V28.8, V28.9

^*^ DRG version 24 was in use from the beginning of the study through 9/30/2007. DRG version 25 was in use from 10/1/2007 through the end of the study.
