# Supplementary material for: Racial disparities and factors associated with pregnancy in kidney transplant recipients in the United States
Source: PLoS One. 2019 Aug 9;14(8):e0220916. doi: 10.1371/journal.pone.0220916 (PMC6688836; doi:10.1371/journal.pone.0220916)
Supplement: S2 Table — (DOCX) [file pone.0220916.s002.docx]

S2 Table. Pregnancy rates with sensitivity analysis performed with conception dates cut off 40 weeks before the end of follow up and removal of women older than 45 years.

|  | **Last 40 weeks deleted** | | **Only time from ages 15-45 included** | |
| --- | --- | --- | --- | --- |
|  | **Unadjusted** | **Adjusted** | **Unadjusted** | **Adjusted** |
| Overall | 13.9 (12.3-15.7) | NA | 15.7 (13.9-17.6) | NA |
| By transplant year |  |  |  |  |
| 2005 | 11.9 (8.3-16.5) | 7.8 (6.6-9.1) | 12.9 (9.1-17.7) | 6.9 (5.8-8.1) |
| 2006 | 15.8 (11.6-21.0) | 9.6 (8.3-11.2) | 16.7 (12.4-22.1) | 8.3 (7.2-9.7) |
| 2007 | 14.1 (10.0-19.2) | 9.3 (7.9-10.9) | 16.3 (11.8-21.8) | 8.8 (7.6-10.3) |
| 2008 | 15.1 (10.6-20.7) | 10.0 (8.5-11.8) | 17.8 (12.9-24.0) | 9.7 (8.3-11.3) |
| 2009 | 14.2 (10.0-19.6) | 9.4 (8.0-11.0) | 17.5 (12.7-23.5) | 9.4 (8.1-11.0) |
| 2010 | 9.9 (6.6-14.3) | 6.4 (5.3-7.7) | 10.4 (6.8-15.2) | 5.4 (4.5-6.6) |
| 2011 | 16.9 (12.2-22.8) | 11.2 (9.6-13.0) | 18.7 (13.6-25.0) | 9.9 (8.5-11.6) |
| By race |  |  |  |  |
| Black | 12.7 (10.0-15.8) | 8.9 (7.9-10.1) | 14.2 (11.3-17.5) | 8.1 (7.2-9.1) |
| Hispanic | 21.0 (16.6-26.2) | 12.4 (10.9-14.0) | 23.9 (19.2-29.5) | 11.6 (10.3-13.1) |
| White | 12.4 (10.1-15.2) | 8.2 (7.3-9.2) | 14.0 (11.4-16.9) | 7.4 (6.6-8.3) |
| Unknown/others | 9.5 (5.3-15.6) | 6.6 (5.2-8.4) | 10.4 (6.0-16.9) | 5.9 (4.7-7.5) |
| By age group (years) |  |  |  |  |
| 15-19 | 22.0 (14.3-32.2) | 20.2 (16.9-24.2) | 24.3 (16.8-33.9) | 22.0 (18.7-25.8) |
| 20-24 | 34.9 (26.2-45.7) | 33.3 (29.3-37.8) | 34.8 (26.5-44.9) | 32.8 (29.1-37.1) |
| 25-29 | 30.7 (24.4-38.1) | 29.4 (26.6-32.6) | 30.2 (24.2-37.2) | 28.6 (25.8-31.6) |
| 30-34 | 18.4 (14.3-23.3) | 18.0 (16.2-20.2) | 17.9 (14.0-22.6) | 17.4 (15.6-19.4) |
| 35-45 | 3.7 (2.7-5.1) | 3.7 (3.2-4.3) | 4.3 (3.0-5.9) | 3.1 (2.7-3.7) |
| By year of conception |  | NA |  | NA |
| Year 1 post-transplant | 9.1 (7.0-11.5) |  | 9.3 (7.2-11.9) |  |
| Year 2 post-transplant | 16.0 (13.2-19.2) |  | 18.4 (15.2-22.2) |  |
| Year 3 post-transplant | 18.2 (14.6-22.5) |  | 20.7 (17.0-24.9) |  |
