# Supplementary material for: Racial disparities and factors associated with pregnancy in kidney transplant recipients in the United States
Source: PLoS One. 2019 Aug 9;14(8):e0220916. doi: 10.1371/journal.pone.0220916 (PMC6688836; doi:10.1371/journal.pone.0220916)
Supplement: S3 Table — (DOCX) [file pone.0220916.s003.docx]

S3 Table. Pregnancy rates with sensitivity analyses performed with removal of deaths and graft failure, and removal of those with missing creatinine.

|  | **Deaths and Graft Failures removed (N=6503)** | | **Missing creatinine removed (N=7437)** | |
| --- | --- | --- | --- | --- |
| **Covariate in Multivariable Logistic Models** | **Odds Ratio (95% CI)** | **P Value** | **Odds Ratio (95% CI)** | **P Value** |
| Age at transplant (years) |  | <0.0001 |  | <0.0001 |
| 15-19 | 0.59 (0.34-1.01) |  | 0.63 (0.39-1.04) |  |
| 20-24 | 1.05 (0.70-1.59) |  | 1.10 (0.74-1.62) |  |
| 25-29 | Reference |  | Reference |  |
| 30-34 | 0.56 (0.38-0.81) |  | 0.70 (0.49-0.99) |  |
| 35-45 | 0.12 (0.08-0.18) |  | 0.14 (0.09-0.21) |  |
| Race |  | 0.0140 |  | 0.0068 |
| Black | 1.07 (0.74-1.54) |  | 0.97 (0.69-1.37) |  |
| Hispanic | 1.53 (1.07-2.18) |  | 1.54 (1.10-2.14) |  |
| White | Reference |  | Reference |  |
| Other | 0.57 (0.28-1.15) |  | 0.65 (0.35-1.21) |  |
| Donor type |  | 0.8628 |  | 0.6162 |
| Living | 1.03 (0.75-1.40) |  | 0.93 (0.69-1.25) |  |
| Deceased | Reference |  | Reference |  |
| Transplant Year | 1.01 (0.94-1.08) | 0.7752 | 1.02 (0.95-1.08) | 0.6454 |
| Cause of end stage kidney disease |  | 0.5801 |  | 0.2764 |
| Cystic/hereditary | 2.13 (0.87-5.23) |  | 2.46 (1.04-5.85) |  |
| Diabetes mellitus | Reference |  | Reference |  |
| Glomerulonephritis/vasculitis | 1.67 (0.80-3.48) |  | 2.15 (1.07-4.32) |  |
| Hypertension/large vessel disease | 1.71 (0.78-3.76) |  | 1.93 (0.91-4.10) |  |
| Miscellaneous | 1.61 (0.75-3.45) |  | 1.95 (0.95-4.04) |  |
| Immunosuppression at time of transplant |  |  |  |  |
| Cyclosporine/tacrolimus | 1.44 (0.70-2.98) | 0.3209 | 1.60 (0.81-3.18) | 0.1797 |
| Mycophenolate | 0.60 (0.36-0.99) | 0.0458 | 0.68 (0.42-1.10) | 0.1129 |
| Sirolimus | 0.89 (0.46-1.69) | 0.7098 | 1.11 (0.62-1.99) | 0.7305 |
| Prednisone/steroids | 0.63 (0.38-1.05) | 0.0759 | 0.67 (0.42-1.08) | 0.1009 |
| GFR at six months after transplantation (ml/min/1.73 m^2^) |  | 0.1299 |  | 0.0634 |
| ≥60 | Reference |  | Reference |  |
| <60 | 0.74 (0.55-1.00) |  | 0.78 (0.59-1.01) |  |
| Missing creatinine value | 0.55 (0.07-4.17) |  | NA |  |
| Duration of dialysis |  | 0.9097 |  | 0.8303 |
| <3 year | Reference |  | Reference |  |
| ≥3 year | 0.98 (0.72-1.34) |  | 0.97 (0.73-1.29) |  |
| Comorbidities |  |  |  |  |
| Diabetes mellitus | 0.99 (0.50-1.98) | 0.9788 | 1.06 (0.55-2.02) | 0.8678 |
| Hypertension | 1.67 (1.18-2.36) | 0.0040 | 1.56 (1.13-2.15) | 0.0063 |
| Congestive heart failure | 1.36 (0.74-2.52) | 0.3219 | 1.59 (0.93-2.72) | 0.0890 |
